# Determinants of underweight among 6–59 months old children in Berahle, Afar, North East Ethiopia: a case control study 2016

**DOI:** 10.1186/s13104-019-4805-z

**Published:** 2019-11-19

**Authors:** Solomon Hintsa, Kiros Gereziher

**Affiliations:** 1 Department of Epidemiology and Biostatistics, School of Public Health, College of Health Science, Aksum University, P. O. Box: 298, Aksum, Ethiopia; 2 Department of Nutrition, School of Public Health, College of Health Science, Aksum University, Aksum, Ethiopia

**Keywords:** Determinants, Underweight, 6–59 months, Berahle, Afar, Ethiopia

## Abstract

**Objective:**

The objective of this study was to identify determinants of underweight among 6–59 months old children in Berahle Woreda, Afar, North East Ethiopia, in 2016.

**Result:**

The median age (IQR) of cases and controls were 24 (34) and 18 (23) months respectively and 51.6% of the children were not exclusively breast-fed but 64.8% controls were exclusively breastfed. Age group of 48–59 months (AOR = 11.93; 95% CI 3.88–36.67), illiterate mothers (AOR = 2.32; 95% CI 1.19–4.55), low dietary diversity (AOR = 4.57; 95% CI 2.40–8.69), diarrhea in the past of 2 weeks (AOR = 2.93; 95% CI 1.46–5.85), birth interval (AOR = 5.17; 95% CI 2.37–11.26) and unprotected source of water (AOR = 2.62; 95% CI 1.42–4.85) were determinant factors of underweight.

## Introduction

Malnutrition is defined as a pathological state resulting from complete, relative deficiency or excess of one or more of the crucial nutrients [1]. It is both medical and social disorder [2].

True underweight may be a sign of dietary, health, or emotional problems [3]. According to the WHO growth standard, underweight in under-five is measured by weight for age and it is the best global indicator of wellbeing in children [4, 5].

Underweight continues as a major public health problem in developing countries. It is the risk factor of the greatest loss of disability-adjusted life years (DALYs [6]). Even though child underweight is a global problem but the magnitude varies [7]. Underweight is highly associated with poverty [8]. In Ethiopia underweight in children is one of the public health problems [9]. In Sub-Saharan Africa (SSA) underweight is contributing to morbidity and mortality in under 5 years [10].

The period vulnerability to nutritional deficiencies is in the early life of pregnancy to 2 years old. Chronic undernutrition in early childhood also results in diminished cognitive and physical development [11, 12].

Half of all deaths in under-five children were attributed to undernutrition. This translates into the unnecessary loss of about 3 million young lives a year [13]. According to the 2013 united nation children’s fund, WHO and the World Bank joint database, globally an estimated 101 million children under 5 years of age or 16% were underweight in 2011 [14]. West and Central Africa have experienced the smallest relative decrease, with an underweight prevalence of 22% in 2014, down from 31% in 1990 [15].

Although the worldwide prevalence of underweight among under-five children has decreased since 1990, still millions of children remain at risk [16]. According to Mini Ethiopian Demographic and Health Survey (MEDHS) 2014 data, overall in Ethiopia 25% of under-five is underweight: prevalence differs across the regions where; in Addis Ababa is 7.2% and in Afar is 45.6% [17, 18].

Common risk factors of undernutrition are low socioeconomic status, low paternal education, lack of sanitation, diarrhea, infections, family size, low birth weight, children age and short birth interval, social and cultural causes, inadequate exclusive breastfeeding, unsuitable complementary feeding, insufficient energy and micronutrient intake, birth order, food availability, housing condition, health services and vaccination status [19–24].

Despite the great focus on child nutrition by the Ethiopian government and partners, underweight is still a public health problem suggesting some factors need to be identified [17]. Therefore, the goal of this study was to identify the determinant factors of underweight among 6–59 months old children.

## Main text

### Study design and area

A case–control study was conducted in Berahle woreda from January 1–March 1/2016. Berahle woreda is found in Afar regional state which is 883 km north of Addis Ababa. The total population of the district is expected to 94,082 based on the census of 2007 where 3575 are under-five children. The district has 04 health centers and 09 health posts. The population are pastoralist community and their life depends on animal production.

### Study population

The source population was all 06–59 months old children living in Berahle woreda. Underweight children were those below − 2 standard deviations and Normal-weight children were those between − 2 and + 2 standard deviations from the median weight-for-age of the WHO/NCHS reference population.

Sample size was determined; by EpiInfo statistical software to determine two-population proportion [25] using 95% CI, Power 80%, control to case ratio 2, OR = 2.3, 38% of exposure among case, 21% of exposure among controls, 10% non-response rate and 1.5 design effect: total calculated sample size was 375 (125 cases and 250 controls). A multi-stage random sampling procedure was used to select study participants. Four Kebeles (smaller administrative units) were selected by simple random sampling from nine Kebeles. A preliminary survey was conducted to differentiate cases and controls in the four Kebeles. We use WHO/NCHS median weight-for-age to identify normal (between − 2 and + 2 SD) and underweight (< − 2 SD) children. According to the survey, 419 cases and 1164 controls identified. Cases and controls were proportionally allocated for each kebele.

### Data collection tools process

Questioner was developed from a standard questionnaire of WHO, EDHS and relevant literature review [17, 25]. The questionnaire was prepared in English and translated to the local language (Afar afe) then retranslated back to English. The questionnaire was pretested in 10% of the total sample size Abala district. Data were collected from mothers or caregivers who had 6–59 months old child by interview and measurement. Four trained health extension workers (community health professionals) were recruited as data collectors and supervised by 2 nurses. Daily supervision was conducted to follow for completeness and consistency of the data.

The content of the questionnaire was socio-demographic, maternal and childcare characteristics, environmental health and dietary history based on 24-h recall.

Anthropometric measurements were measured using the standard technique. Weight of the child and mothers or caregivers were measured with light clothing and without shoes using UNICEF Electronic Scale with an accuracy of 0.1 kg. The mother was weighed with the child and weigh without the child and then subtract the weight of the mother from the total weight to get the weight of the child [25]. Height was measured using Stadiometer (Seca, Germany) in centimeter (cm) in an erect position that the back of the head, shoulder blades, buttocks, and heels make contact with the backboard at a precision of 0.1 cm.

### Data analysis and management

Collected data were cleaned, edited, coded and entered into Epi data software version 3.1 and exported to SPSS version 20. Descriptive statistics were presented in tables. Variables with P-value < .25 in Bivariable logistic regression were taken to multiple Logistic regression. A backward likelihood ratio with 0.1 probability removal was used to develop the model. OR was estimated with 95% CI to show the strength of association and P-value < .05 was used to Declare statistical significance. The goodness of fit of the final model was checked using the Hosmer–Lemeshow test of goodness at P-value ≥ .05 (.135), model classification of accuracy was checked with 82.6% correctly classified and multicollinearity was assessed using standard errors of the Beta coefficients at > 2.

### Ethical consideration

The study has received ethical approval from Mekelle University, College of Health Science Ethical Review Board before commencement. Permission letter was obtained from Afar regional health bureau and Berahle woreda health office.

### Results

A total of 368 (124 cases and 244 controls) with a 98.1% response rate participated in this study. Of cases, 34 (27.4%) were in the age group of 12–23 months while 80 (32.8%) controls were 6–11 months age group. All (100%) children were ever breastfed and 64 (51.6%) of the children were not exclusively breastfed but 158 (64.8%) controls were exclusively breastfed (see Table 1).

**Table 1:** Cross tabulation of socio demographic characteristics of study participants with underweight status among 6–59 months old children in Berahle woreda, 2016 (n = 368)

Variable	Category	Underweight status of the children		
		Yes	No	Total
Age of the children	6–11 months	20 (16.13)	80 (32.79)	100 (27.2)
	12–23 months	34 (27.42)	64 (26.23)	98 (26.6)
	24–35 months	20 (16.13)	46 (18.85)	66 (17.9)
	36–47 months	18 (14.52)	38 (15.57)	56 (15.2)
	48–59 months	32 (25.80)	16 (6.56)	48 (13)
Residence	Rural	79 (63.7)	156 (63.9)	235 (63.9)
	Urban	45 (36.3)	88 (36.1)	133 (36.1)
Marital status	Married	100 (80.6)	200 (82)	280 (76.1)
	Formerly married	20 (16.2)	34 (13.9)	54 (20.1)
	Single	4 (3.2)	10 (4.1)	14 (3.8)
Religion	Muslim	120 (96.8)	210 (86.1)	330 (89.7)
	Orthodox	4 (3.2)	34 (13.9)	38 (10.3)
Husbands occupation	Farmer	52 (41.9)	65 (26.6)	117 (31.8)
	Daily laborer	4 (3.2)	12 (4.9)	16 (4.3)
	Employed	28 (22.6)	87 (35.7)	115 (31.2)
	Merchant	32 (25.8)	70 (28.7)	102 (27.7)
	Other	8 (6.5)	10 (41.1)	18 (4.9)
Educational status of mother	Illiterate	85 (68.5)	134 (54.9)	219 (59.5)
	Literate	39 (31.5)	110 (45.1)	149 (40.5)
Educational status of father	Illiterate	44 (35.5)	90 (36.90	134 (36.4)
	Literate	80 (64.5)	154 (63.1)	234 (63.6)
Income	< 750	24 (19.4)	37 (15.2)	61 (16.5)
	750–1500	68 (54.8)	121 (49.6)	189 (51.3)
	> 1500	32 (25.8)	86 (35.2)	118 (32.2)
Livestock ownership	Yes	72 (58.1)	134 (54.9)	206 (56)
	No	52 (41.9)	110 (45.1)	162 (44)
Decision maker	Husband	33 (26.6)	66 (27)	99 (26.9)
	Wife	14 (11.3)	36 (14.8)	50 (13.6)
	Jointly	77 (62.1)	142 (58.2)	219 (59.5)
Family size	≤ 4	30 (24.2)	72 (29.5)	102 (27.7)
	> 4	94 (75.8)	172 (70.5)	266 (72.3)
Number of < 5 children in the house	One	66 (53.2)	120 (49.2)	186 (50.5)
	Greater one	58 (46.8)	124 (50.8)	182 (49.5)
Sex	Male	68 (54.8)	132 (54.1)	200 (54.3)
	Female	56 (45.2)	112 (45.9)	168 (45.7)
Birth weight (N = 28)	≥ 2500 g	4 (50)	18 (90)	22 (78.6)
	< 2500 g	4 (50)	2 (10)	8 (23.4)
Size of the child during birth (n = 340)	Normal	36 (30.5)	160 (72.1)	196 (57.6)
	Small	80 (67.8)	42 (18.9)	122 (35.9)
	Big	2 (1.7)	20 (9)	22 (6.5)
Birth order	1	18 (14.5)	34 (13.5)	52 (14.1)
	2–4	34 (27.4)	118 (48.4)	152 (41.3)
	> 4	72 (58.1)	92 (37.7)	164 (44.6)
Diarrhea	No	78 (62.9)	214 (87.7)	292 (79.3)
	Yes	46 (37.1)	30 (12.3)	76 (20.7)
Cough	No	111 (89.5)	218 (89.3)	329 (89.4)
	Yes	13 (10.5)	26 (10.7)	39 (10.6)
Fever	No	98 (79)	202 (82.8)	300 (81.5)
	Yes	26 (21)	42 (17.2)	68 (18.5)
Ever breast fed	Yes	124 (100)	244 (100)	368 (100)
	No	0 (0)	0 (0)	0 (0)
EBF	No	64 (51.6)	86 (35.2)	150 (40.8)
	Yes	60 (48.4)	158 (64.8)	218 (59.2)
Duration of EBF (n = 198)	≥ 6 months	14 (35)	79 (48.1)	90 (45.5)
	< 6 months	26 (65)	82 (51.9)	108 (54.5)
Beginning of CF	≥ 6 months	61 (49.2)	128 (52.5)	189 (51.4)
	< 6 months	63 (51.8)	116 (47.5)	179 (48.6)
Cessation of BF (n = 224)	> 24 months	54 (61.4)	98 (72.1)	152 (67.9)
	< 24 months	26 (29.5)	36 (26.5)	62 (27.7)
	24 months	8 (9.1)	2 (1.5)	10 (4.5)

Of participants, 154 (63.1%) controls were meet the minimum dietary diversity score but 84 (67.7%) cases were not meet the minimum dietary diversity score (see Table 2).

**Table 2:** Cross tabulation of child caring practice, maternal, environmental, and dietary diversity characteristics with underweight status among 6–59 months old children in Berahle woreda, Afar, Northern East Ethiopia, 2016

Variable	Category	Underweight status of the children		
		Yes	No	Total
Minimum dietary diversity	Meet	40 (32.3)	154 (63.1)	194 (52.7)
	Not meet	84 (67.7)	90 (36.9)	174 (47.3)
MMF (n = 198)	Not accepted	29 (53.7)	82 (56.9)	111 (56.1)
	Accepted	25 (46.3)	62 (43.1)	87 (43.9)
BCG vaccination	Yes	31 (25)	72 (29.5)	103 (27)
	No	93 (75)	172 (70.5)	265 (73)
Measles vaccination status (n = 310)	Vaccinated	77 (67.5)	123 (62.8)	200 (64.5)
	Not vaccinated	37 (32.5)	73 (37.2)	110 (35.5)
Mothers age	15–24	25 (20.2)	66 (27)	91 (24.7)
	25–34	52 (41.9)	114 (46.7)	166 (45.1)
	35–44	37 (29.8)	60 (24.6)	97 (26.4)
	> 44	10 (8.1)	4 (1.6)	14 (3.8)
BMI	Underweight	60 (48.4)	36 (14.8)	96 (26.1)
	Healthy weight	60 (48.4)	204 (83.6)	264 (71.7)
	Overweight	4 (3.20	4 (1.6)	8 (2.2)
Parity	1	18 (14.5)	42 (17.2)	60 (16.3)
	2–3	33 (26.6)	105 (43)	1381 (37.5)
	> 3	73 (58.9)	97 (39.8)	170 (46.2)
Birth interval (n = 322)	≥ 3	16 (15.1)	92 (42.6)	108 (33.5)
	< 3	90 (84.9)	124 (57.4)	214 (66.5)
Source of water	Protected	60 (48.4)	180 (73.8)	240 (65.2)
	Not protected	64 (51.6)	64 (26.2)	128 (34.8)
Latrine availability	No	82 (66.1)	94 (38.5)	176 (47.8)
	Yes	42 (33.9)	150 (61.5)	192 (52.2)
Hand washing before food preparation.	No	6 (4.8)	12 (4.9)	18 (4.8)
	Yes	118 (95.2)	232 (95.1)	358 (95.2)
Hand washing before &after feeding	No	10 (8.1)	16 (6.6)	26 (7)
	Yes	114 (91.9)	228 (93.4)	342 (93)
Hand washing after toilet	No	41 (33.1)	72 (29.5)	113 (30.7)
	Yes	83 (66.9)	172 (70.5)	255 (69.3)
Hand washing after child cleaning	No	62 (50)	121 (49.6)	183 (49.7)
	Yes	62 (50)	123 (50.4)	185 (51.3)
Cereal	Yes	124 (100)	230 (94.2)	354 (96.1)
	No	0	14 (5.8)	14 (3.9)
Legume and nut	Yes	86 (65.6)	136 (57.4)	222 (60.3)
	No	45 (34.4)	101 (42.6)	146 (39.7)
Dairy product	Yes	84 (64)	178 (75)	262 (71.1)
	No	47 (36)	59 (25)	106 (28.9)
Flesh food	Yes	28 (21.3)	84 (35.4)	112 (30.5)
	No	103 (78.7)	153 (64.6)	256 (69.5)
Egg	Yes	6 (4.6)	36 (15.2)	42 (11.5)
	No	125 (95.4)	201 (84.8)	326 (88.5)
Vitamin A rich fruit and veg	Yes	14 (10.7)	48 (20.3)	62 (16.9)
	No	117 (89.3)	189 (79.7)	306 (83.1)

In multivariable logistic regression, variables with P-value < .25 in binary logistic regression like age of the mother and child, educational level of mother, father’s occupation, mother’s occupation, monthly income, exclusive breastfeeding status, dietary diversity score, body mass index of mothers, birth interval, diarrhea, source of dirking water, faeces disposal system and latrine ownership were included into the model. Finally, the age of the child, Educational status of the mother, birth interval, diarrhea, dietary diversity score and source of drinking water were statistically significant predictors of underweight.

Being in the age group 48–59 months was 11.93 times (AOR = 11.93; 95% CI 3.88–36.67) having underweight than children in the age group of 6–11 months. Illiterate mothers were 2.3 times (AOR = 2.32; 95% CI 1.19–4.55) at high risk of underweight compared to literate mothers. Children who got low dietary diversity score were 4.5 (AOR = 4.57; 95% CI 2.404–8.69) times more likely to become underweight than children who got the minimum acceptable dietary diversity. Children who had diarrhea in the past 2 weeks before data collection were 2933 times (AOR = 2.933; 95% CI 1.469–5.854) higher odds of underweight than children without the diarrheal disease. Short interval (< 3 years) from the mother’s previous childbirth was nearly five times more likely to have an underweight child than their counterpart with (AOR = 5.17; 95% CI 2.37–11.26). Children’s families who used drinking unprotected water sources were 2.6 times more likely to have underweight children compared to those children whose families used drinking protected water sources with (AOR = 2.626; 95% CI 1.42–4.85) (see Table 3).

**Table 3:** Bi variate and multivariable analysis participants on determinants of underweight among 6–59 months old children in Berahle, Afar, Northern East Ethiopia, 2016 (n = 368)

Categories	Case (124)	Control (244)	COR (95% CI)	AOR (95% CI)	P-value
Mothers occupation					
House wife	74 (59.7)	124 (50.8)	1		
Others	50 (40.3)	120 (49.2)	.69 (.45–1.08)		
Fathers occupation					
Farmer	70 (56.5)	99 (40.6)	1		
Employed	20 (16.1)	72 (29.5)	.39 (.22–.70)		
Merchant	22 (17.7)	51 (20.9)	.61 (.33–1.09)		
Other	12 (9.7)	22 (9)	.77 (.35–1.66)		
Mothers literacy					
Illiterate	85 (68.5)	134 (54.9)	1.78 (1.13–2.82)	2.32 (1.19–4.55)*	.014
Literate	39 (31.5)	110 (45.1)	1		
Income					
< 750	24 (19.4)	37 (15.2)	1.74 (.90–3.35)		
750–1500	68 (54.8)	121 (49.6)	1.51 (.913–2.49)		
> 1500	32 (25.8)	86 (35.2)	1		
Age of children					
6–11 months	20 (16.1)	80 (32.8)	1		
12–23 months	34 (27.4)	64 (26.2)	2.12 (1.11–4.04)	1.03 (.41–2.53)	.948
24–35 months	20 (16.1)	46 (18.9)	1.74 (.84–3.56)	1.71 (.66–4.41)	.265
36–47 months	18 (14.5)	38 (15.6)	1.90 (.90–3.99)	1.77 (.69–4.56)	.231
48–49 months	32 (25.8)	16 (6.8)	8 (3.68–17.36)	11.93 (3.88–36.67)*	.000
Birth order					
1	18 (14.5)	42 (17.2)	1		
2–3	33 (26.6)	105 (43)	.73 (.37–1.44)		
> 3	73 (58.9)	97 (39.8)	1.70 (.93–3.29)		
Diarrhea					
No	78 (62.9)	214 (87.7)	1		
Yes	46 (37.1)	30 (12.3)	4.2 (2.48–7.13)	2.93 (1.46–5.85)*	.002
EBF status					
No	64 (51.6)	86 (35.2)	1.96 (1.26–3.04)		
Yes	60 (48.4)	158 (64.8)	1		
EBF duration					
≥ 6 months	14 (35)	79 (48.1)	1		
< 6 months	26 (65)	82 (51.9)	1.72 (.83–3.53)		
Score diet diversity					
Meet	40 (32.3)	154 (63.1)	1		
Not meet	84 (67.7)	90 (36.9)	3.59 (2.27–5.67)	4.57 (2.40–8.69)*	.001
Source of water					
Protected	60 (48.4)	180 (73.8)	1		
Not protected	64 (51.6)	64 (26.2)	3 (1.90–4.7)	2.62 (1.42–4.85)*	.002
Latrine ownership					
No	82 (66.1)	94 (38.5)	3.11 (1.98–4.89)		
Yes	42 (33.9)	150 (61.5)	1		
Faeces disposal system					
Proper disposal	56 (45.2)	131 (53.7)	1		
Improper disposal	68 (54.8)	113 (46.3)	.71 (.46–1.09)		
Body mass index					
Underweight	63 (50.8)	83 (34)	2 (1.29–3.11)		
Normal weight	61 (49.2)	161 (66)	1		
Birth interval					
≥ 3	16 (15.1)	92 (42.6)	1		
< 3	90 (84.9)	124 (57.4)	4.17 (2.29–7.57)	5.17 (2.37–11.26)*	.000

*AOR* adjusted odds ratio, *CI* confidence interval

* Significant predictors at P-value < .05. Variables with P-value < .25 in the bivariable analysis (COR) were included to the final model for adjustment (AOR)

### Discussion

The result of this study revealed that the age of the child, educational status of the mother, birth interval, diarrhea, dietary diversity and source of drinking water were statistically associated determinant factors of children underweight.

The age group of 48–59 months was 11.93 times high odds of underweight than children in the age group of 6–11 months. This study is consistent with a study from Lalibela where children aged from 48 to 59 months were about 2.9 times more likely to be underweight than children 6–11 months old. The difference in the strength of association may be due to study design. Studies done from Ghana, Egypt and Tigray [21, 26, 27] revealed that children in 12–23 and 23–35 months were highly affected by underweight. This might be explained by the fact that 48 to 59 months old children are more active and lose a greater amount of energy that makes them underweight. Besides, mothers or caregivers may give more care for the new births.

Illiterate mothers were at higher risk of having underweight children. Studies conducted in Somali, Brazil, Arba Minch, Gondar, Nepal, Nigeria, and Tanzania have a consistent result with this study [28–34]. Being illiterate mothers were 3.28 times and 1.4 times at high risk for underweight in Brazil [29] and Arba Minch [30] respectively. This slight difference in the strength of association may be due to study design, sample size and geographical location. It seems plausible that the educational status of the mother affects awareness of exclusive breastfeeding and complementary feeding practice and thus could affect their weight status.

Diarrhea was also a significant determinant of underweight. Children with diarrhea in the past 2 weeks were 2.933 times more likely to be underweight than those who had no diarrhea. Even though there is a slight difference in the strength of association among many studies, the finding of this study was consistent with the following cited researches [21, 35–38]. This might be due to less health care support, poor hygiene, and sanitation.

The source of drinking water is also a major determinant of underweight in this study. This is supported by studies done in Ethiopia [23]. Nigeria [39] and Brazil [29]. The possible explanation might be due to access to clean water reduces the chance of exposure of the child to water-borne diseases like diarrhea.

Another determinant variable for this study was a dietary diversity score where children who were not meet minimum dietary diversity score were 4.57 times more likely to become underweight. Similar studies were also seen in Nekemt [40], Arba Minch [30] and Teheran [41]. This might be due to diversified food reflects higher dietary quality and a greater possibility of meeting the daily energy and nutrient requirements of children.

Short interval from mother’s previous birth was a risk factor for underweight. The result of this study is supported by studies done in Teheran [41, 42]. This might be from its effect through decreasing birth weight and children born within 3 years of a sibling are generally prone to inadequate care from their mothers.

### Conclusion

This study identifies multiple risk factors for child underweight status. Being in Age group of 48–59 months, being illiterate, less than 3 years of birth interval, diarrhea, not meet minimum dietary diversity score and using an unprotected source of drinking water were statistically significant determinants of underweight. The varieties of risk factors identified in this study emphasize the need for a multidisciplinary approach to address the issues of child underweight. Women development army, community health workers, Woreda health office, and NGOs like Goal Ethiopia and Woreda water supply office should emphasize the determinant factors.

## Limitations

There may also some limitations in the study like; Recall bias and inaccurate responses like age and the nutritional factors.

## Data Availability

The data sets used and analyzed during the current study available from the corresponding author on reasonable request.

## Abbreviations

AOR: adjusted odd ratio
BMI: body mass index
CI: confidence interval
COR: crude odd ratio
DALY: disability-adjusted life year
MEDHS: Mini Ethiopian Demographic Health Survey
IQR: inter quartile range
OR: odd ratio
SDG’s: sustainable developmental goals
SPSS: statistical packaging for social science
SSA: Sub Saharan Africa
UNICEF: United Nations Children’s Fund
WHO: World Health Organization
